# Determining workload and size of nursing team in the pediatric emergency department

**DOI:** 10.1590/S1679-45082014AO2945

**Published:** 2014

**Authors:** Ana Cristina Rossetti, Raquel Rapone Gaidzinski, Mario Maia Bracco

**Affiliations:** 1 Sociedade Beneficente Israelita Brasileira Albert Einstein, Hospital Municipal Dr. Moysés Deutsch – M'Boi Mirim, São Paulo, SP, Brazil; 2 Escola de Enfermagem, Universidade de São Paulo, São Paulo, SP, Brazil

**Keywords:** Workload, Nursing staff, hospital, Personnel administration, hospital

## Abstract

**Objective:**

To identify indicators of required nursing workload for pediatric patients care in an emergency department.

**Methods:**

This cross-sectional quantitative study was carried out in a pediatric short-stay unit at a public hospital in São Paulo (SP), Brazil. The patient classification system and activity times of nursing care proposed by the Brazilian Federal Nurse Council were applied to all patients admitted over a 1-month period. The instruments were applied by two nurses in populations of 500 and 453 patients, respectively. Nursing workload was calculated in terms of hours and by nurse/patient ratio. Data were analyzed by descriptive statistics, and inter- and intraobserver reliability was assessed by using Kappa coefficient. Confidence intervals were measured to verify differences in numbers of patient among work shifts.

**Results:**

The average daily workload was 108.7 hours and the average amount of time spent on nursing care per patient was 7.9 hours. The nurse-patient ratio was 1:2.6.

**Conclusion:**

The application of the standardized classification instrument was adequate. It enabled the classification of pediatric patients and the measurement of amount of time needed for nursing care. The average number of nursing work hours per patient met the average time for semi-intensive care established by the Brazilian Federal Nurse Council.

## RESUMO

**Objetivo:**

Identificar indicadores de carga de trabalho de enfermagem necessária ao atendimento de pacientes pediátricos em um prontosocorro geral.

**Métodos:**

Pesquisa transversal, quantitativa, realizada na área de observação de pacientes pediátricos em hospital público, no município de São Paulo (SP). Foi aplicado o Sistema de Classificação de Pacientes e os tempos de assistência de enfermagem, propostos pelo Conselho Federal de Enfermagem, em todos os pacientes internados no período de 1 mês, por dois enfermeiros em 500 e 453 pacientes, respectivamente. A carga de trabalho foi calculada em horas e pela razão entre enfermagem e pacientes. Os dados foram analisados por estatística descritiva e análise de concordância de Kappa entre os observadores e com o instrumento. Foram calculados os intervalos de confiança para verificar as diferenças no número de pacientes entre os turnos de atendimento.

**Resultados:**

A carga média diária de trabalho correspondeu a 108,7 horas e o tempo médio de assistência de enfermagem por paciente foi 7,9 horas. A relação enfermagem-paciente foi de 1:2,6.

**Conclusão:**

A aplicação de instrumento de classificação padronizado mostrou-se adequada e possibilitou a classificação dos pacientes pediátricos, além do dimensionamento do tempo de cuidado necessário. O tempo médio de trabalho da equipe de enfermagem por paciente correspondeu ao tempo médio de cuidado semi-intensivo estabelecido pelo Conselho Federal de Enfermagem.

## INTRODUCTION

Nursing workload at hospital is related to care needs of patients and to standard of care intended,^(1)^. A range of factors and the size of professional team contribute to workload.^(2)^

Carayon and Alvarado^(3)^ described seven interrelated dimensions associated with workload that involved quantitative and qualitative aspects such as patient care, working under temporal constraint, dealing with emotional issues and variability of the clinical picture. For this reason, the adequate number of nursing staff can create conditions to optimize workload. The sub-dimensioning of staff increases workload and the impacts toward all dimensions in a cyclic manner, may to lead to compromised quality of care and affects the safety of patient and nursing professionals.^(2,3)^

Studies on nursing workload in different types of hospital units^(4-7)^ have shown that the use of patient classification system (PCS) to determine the degree of dependence of the patient in nursing care enables to measure the amount of nursing workload needed for the care of patients with different ages.^(8-10)^

## OBJECTIVE

Because of the scarcity of studies related to workload in observation areas of pediatric emergency and urgent care services the aim of this study was to determine nursing workload indicators required for pediatric patients care in observation areas at a general emergency department.

## METHODS

This population based, cross-sectional and quantitative study was carried out from June 8 to August 6 2010 in an observation area of pediatric patients (OAP) at the emergency department of the Hospital Municipal Dr. Moysés Deutsch – M'Boi Mirim (HMMD) in the south region of São Paulo city.

The HMMD is classified as a large secondary hospital in the Brazilian Health Service,^(11)^ which is reference for a population of 600,000 individuals of the south region of São Paulo.

The OAP in the emergency department is designed for follow-up of short-stay pediatric patients. The patients at this area can be discharged or referred to hospitalization depend on their clinical picture. This department has 22 beds, and of these, 2 for quarantine and 1 for emergency.

The HMMD, including the OAP, has adequate nursing practices and nursing process based on nursing care systematization and quantitative dimensioning of multidisciplinary team previously established considering patient load since its foundation in 2008.^(12)^

Workload was measured in hours and by nursing-patient ratio. Beds in the OAP were classified according to PCS proposed by Dini et al.^(9)^ and care categories by Fugulin et al.,^(13)^ who considered intensive care for pediatric patients at any age, whose condition is clinically unstable, without imminent risk of death and require continuing and specialized nursing and medical care; semi-intensive care for pediatric patients at any age, whose condition is clinically unstable, without imminent risk of death and require continuing and specialized nursing and medical care; high dependency care for pediatric patients at any age whose condition is clinically stable and have physical, emotional and social needs which require nursing care; intermediate care for pediatric patients aged 7 years or older with development compatible for their age, whose condition is clinically stable and require nursing care for self-care and/or support to deal with the disease and hospitalization; and minimal care for pediatric patients aged 12 years or older with development compatible for their age, whose condition is clinically stable and are able to perform their self-care activities under the supervision of nursing team.

Time spent on nursing care in each duty established in PCS proposed by Dini et al.^(9)^ were proposed by the Brazilian Federal Nurse Council (COFEN, acronym in Portuguese) in the resolution 293/04.^(14)^ It reinforced the PCS stated by Fugulin et al.^(8)^ – excepted the high dependency care. The resolution stated that in terms of measurement of nursing staff, the hours spent by nurses in each bed for 24 hours must be as follow: 8.8 hours for minimal nursing care or self-care; 5.6 hours for intermediate nursing care; 9.4 hours for semi-intensive nursing care; 17.9 hours for intensive nursing care.

For patients classified as high dependent, whom time of care was not established by CONFEN resolution 293/04,^(14)^ we used the same nursing care hours indicated for semi-intensive patients. Perroca and Gaidzinski^(10)^ classified high dependent patients as semi-intensive chronic patients so that attributing 9.4 hours per patient for every 24 hours.

The average daily workload required by patients was measured by the sum of product from the average number of occupied bed daily for each care category 
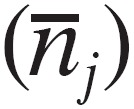
 and the average time for nursing care spent in each care category 
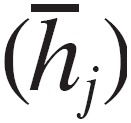
, which was defined by COFEN Resolution 293/04^(14)^. The equation 1 was used for the measurement.

(Equation 1)
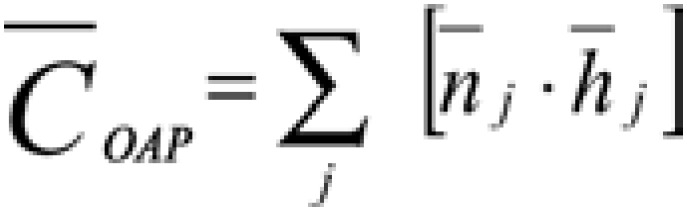


In which:


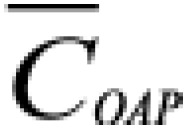
 = average daily workload in the pediatric observation area;


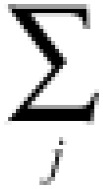
 = sum of average daily workload related to each care category of PCS _j_;

j = any type of care;


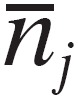
 = average number of occupied beds daily in OAP related to each care category of PCS _j_;


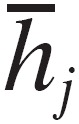
 = average time of care in OAP in each category of PCS _j_.

The relationship nursing-patient was measured using the division between average time spent in care per patient and activity time within 24 hours of 85%,^(15)^ considering studies that revealed that roughly 15% of time spent by nursing professionals was to personal needs.^(5,6,16)^ Levels higher than 90% can represent increase in costs and decrease in care quality and in nursing results.^(16)^ This relationship was seen in equation 2:^(15)^

(Equation 2)
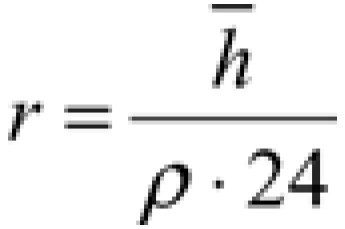


In which:

r = relationship nursing-patient;


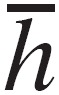
 = average care time needed for each patient;

ρ = time of production.

Data were collected between June 8 and August 6, 2010 once a day, every day at 8 AM in occupied beds independently of length of stay. Because length of stay and time of admission in OAP varied, to assure that admission routines and discharge did not interfere in workload verified at 8 AM, the number of patients was checked at four times during the day: 8 AM, 2 PM, 8 PM and 2 AM that corresponded to work shifts in the morning (7 AM to 1 PM), afternoon (1 to 7 PM) night 1 (7 PM to midnight) and night 2 (midnight to 7AM).

Data were collected by two nurses with experience in the area and who were previously trained. The trainings included reading assignments, discussion and practical application of the instrument items with the aim to create a standard for comprehension and ensure the standard in forms filling during data collection.

Reliability of patient classification was verified by concordance test for the data collected between the two nurses and of these nurses concerning to description of care category indicated by the instrument by Dini et al.^(9)^

In case of disagreement in instruments, nurses were instructed to document care category based on perception that best classified the child. The concordance of these perceptions between the two nurses and related to the instrument by Dini et al.^(9)^ were also analyzed.

Data were analyzed using descriptive statistics and Kappa coefficient between observers and of each of them with the instrument. Confidence interval was measured to verify differences in the number of patient in morning, afternoon, nigh 1 and night 2 work shifts. We adopted p<0.05 and the analyses were carried out using the Statistical Package for Social Science version 17.0 (SPSS Inc., Chicago, III).

This study was approved by the Ethical and Research Committee of the *Sociedade Beneficente Israelita Brasileira Albert Einstein* (process CEP/Einstein nº 10/1287, CAAE: 0021.0.028.196-10). Consent form was not required because no human subjects were involved.

## RESULTS

Nurse 1 and nurse 2 collected data from populations of 500 and 453 patients, respectively. All patients evaluated by nurse 2 were also evaluated by nurse 1.

Patients mean age was 3.1 years (standard deviation=3.7). The youngest child age was 0 (less than one month of age) and oldest child age was 13 years.

There were significant differences between work shifts. The night 2 and morning shifts presented the largest number of patients (Table 1).

**Table 1:** Number of patients per work shift

OAP	Night 2 (2h)	Afternoon (14h)	Afternoon (14h)	Night 1 (20h)
Mean (SD)	10.9 (4.1)*	11.1 (3.5)**	9 (4.3)* ^,^ **	8.3 (3.7)* ^,^ **
Medium (p25 - p75)	10 (8-13)*	11 (9-14)**	9 (6-11)* ^,^ **	8 (6-9.5)* ^,^ **
Minimal-Maximal	4-22	4-19	1-20	2-23

* p<0,05 night 2 *versus* afternoon and night 1;

** p<0,05 morning *versus* afternoon and night 1.

OAP: observation area of pediatric patients; SD: standard deviation.

**Figure 1:**
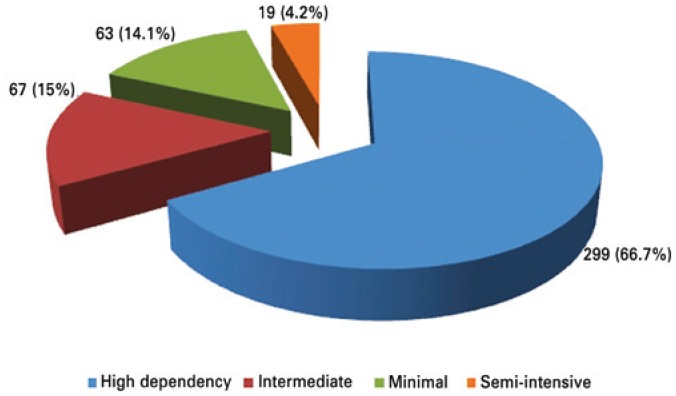
Concordance in care categories between nurses during observation of pediatric patients

A strong concordance was observed related to perceptions of care category between the two nurses with Kappa coefficient of 0.98. This represented a concordance in 448 patients (Figure 1).

The two nurses agreed 100% concerning care category documentation, when they applied the instrument,^(9)^ and in the category description. The Kappa coefficient of nurses' perception related to instruments was 0.41 and for nurses 1 and 0.42 for nurses 2. The lowest concordance occurred in intermediate care category being 30.4 and 31.6% of concordance for nurses 1 and 2, respectively. The concordance related to possibility of children classified as intermediary care to be classified as minimal care was 62 and 60.5% for nurse 1 and 2, respectively. The best concordance was seen in the semi-intensive care category in which both nurses agreed 100% followed by category of high dependency care presenting 82.6% and 81.9% for nurse 1 and nurse 2, respectively.

On average distribution of patients for type of care per day was also grouped according to concordance of nurses' perception (Table 2).

**Table 2:** Distribution of patients by type of care based on concordance of nurses' perception

	Type of care				
	Intensive	Semi-Intensive	High dependency	Intermediate	Minimal
Mean (SD)	–	1.9 (1)	7.5 (2.8)	2.2 (1.3)	2.1 (1.2)
Medium	–	2	7	2	2
Minimal-Maximum	–	1-4	2-14	1-5	1-5

SD: standard deviation.

The average daily workload, which was measured in hours, was calculated using the hours recommended by COFEN resolution 293/04^(14)^ and replacing the values of 
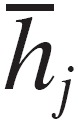
 and 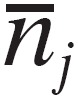 of equation 3, which are proposed for this area, as following:

(Equation 3)
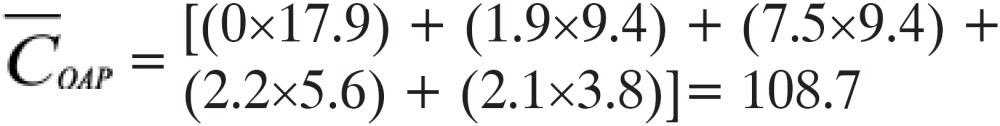


The average daily workload was 108.7 hours. The average amount of time spent on nursing care per patient was 7.9 hours, considering the average of 13.7 patients.

To establish the relationship nursing-patient in the area and convert it in decimal number, the equations 4 and 5 were used, and the relationship nursing-patient found was 1:2.6:

(Equation 4)
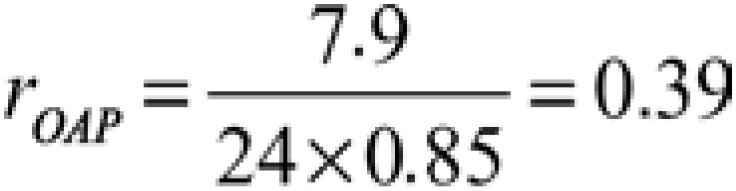


(Equation 5)
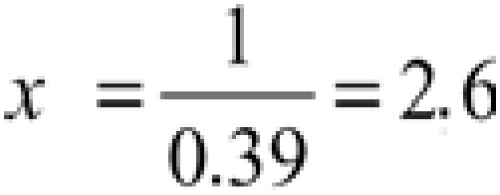


## DISCUSSION

This study verified nursing workload needed for the care of pediatric patients in the observation area of the emergency department at general public hospital. The application of the standard classification instrument was effective to determine both dependence degree and size of nursing staff according to COFEN resolution 293/04.^(14)^ To date, there are no reports in the literature on the use of patient classification instrument by Dini et al.^(9)^ associated with OAP. The use of this instrument enabled the classification of pediatric patients and the measurement of amount of time needed for nursing care.

The size of the nursing staff is directly associated with competence and experience of professionals as well as with degree of dependence and risks related to patients' clinical conditions.^(17)^ Instruments to measure the degree of dependence among patients must be valid and reliable to guarantee patient safety and quality in nursing care. In a review of the literature with 12 studies that measured this issue in emergency services, only 3 studies had evidence of good validity and reliability.^(18)^

In our study, the reliability test of instrument showed that concordance of nurses concerning category description of care indicated by the instrument by Dini et al.,^(9)^ showed that the instrument is ease to be applied by experienced professionals.

When the reliability test was applied, considering the perception of the nurse 1 and 2 related to description of care category of the instrument, the medium concordance was^(19)^ 0.41 and 0.42, respectively, and among these professionals, the concordance was excellent (0.98). Based on perception of nurse 1 and 2, 62% and 60.5% of children were classified as requiring minimal care, respectively. It is relevant to consider the experience of professionals to deal with pediatric patients and also their perception related to the degree of dependence of these patients.^(20)^ Another important consideration is to understand the socioeconomic profile of the population assisted because poverty status might lead to higher independence of the child mainly in girls aged 10 years or older.

Level of concordance between nurse 1 and 2 was excellent for the categories semi-intensive care (100% for both nurses), high dependence (82.6% and 81.9%, respectively) and minimal (94.1% and 100%, respectively). The lowest concordance of nurses' perception was seen in intermediary care category. Perroca and Gaidizinski,^(21)^ in a study of concordance between nurses related to category of intensive care unit concluded that it seems to be easy for nurses to classify patients in category of intensive and minimal care because opposite ideas are easily identified by presence or absence of factors that evidence the severity in adult patients.

Concordance in classification of patient care category based on nurses 1 and 2 perception was 0.98. It was considered excellent. This result shows that the same understand was achieved concerning the instrument application and concerning care categories of the instrument. In a study by Williams e Crouch^(18)^ on validation and reliability of PCS for adult in six emergency services in United Kingdom reported a Kappa coefficient of 0.75.

The average number of nursing work hours per patient was 9.8 hours. This result meets the average time for semi-intensive care established by the COFEN resolution 293/04.^(14)^

A limitation of this study was the classification of degree of dependence by nurses for 8 hours only, although the number of patients in the other shifts was documented. The difference in number of patients among work shifts, which was higher on the morning and on night 2, can be related to shifts managements such as exchanges in medical shifts on 7 PM and 7 AM, a period that assessment and discharges can be more frequent. In addition, municipality emergency services in the area where the hospital analyzed is located open only during the day, and the HMDD is unique service opened 24 hours/daily. Williams and Crouch reported no correlation between number of patients per work shifts and degree of dependence.^(18)^

Although validation of the instrument was not the goal of our study, the moderate values found in Kappa coefficient concerning care categories suggested that the instrument can be improved, so that, further validation studies related to clinical outcomes with larger samples are warranted.^(22)^ Currently, there is no studies in the literature on validation of classification systems for pediatric patients in emergency services.

It is not possible to generalize the results of this study because of particularities of the HMMD and the region where the hospital is located. However, because of the scarcity of resources and infrastructure deficiencies of the Brazilian Health System, this study's findings can contribute to improve understands on improvement required in the management and financing of the Brazilian public health.

## CONCLUSION

This is the first study on the amount of nursing workload needed for observation of pediatric patients. The instrument used was effective to determine the workload and size of nursing staff according to the degree of dependence of pediatric patients.

In context of Brazilian public hospitals, mainly in large urban centers where need for health services is great and such services are limited, there is an evidence of the overload of existed services. The improvement of efficiency in observation of short-stay pediatric patients brings benefits to patients, families, professionals and to the health system that can lead to high level of satisfaction and reduction in costs.

Further studies are warranted to assess activities of nurses during observation of pediatric patients as well as the validation and reliability of instruments to standardized pediatric patients classification in order to improve the understand of the dynamic of the work and enhance the quality of care delivery by nursing teams.
